# Cervical schwannoma in the early stage of pregnancy: a case report

**DOI:** 10.1186/s12893-020-00903-8

**Published:** 2020-10-20

**Authors:** Koki Kawaguchi, Koji Akeda, Norihiko Takegami, Tatsuya Kurata, Kuniaki Toriyabe, Tomoaki Ikeda, Akihiro Sudo

**Affiliations:** 1 Department of Orthopaedic Surgery, Mie University Graduate School of Medicine, 2-174 Edobashi, Tsu, Mie 514-8507 Japan; 2 Department of Orthopaedic Surgery, Sakakibara Onsen Hospital, 1033-4 Sakakibara town, Tsu, Mie 514-1293 Japan; 3 Department of Obstetrics and Gynecology, Mie University Graduate School of Medicine, Mie, Japan

**Keywords:** Spinal schwannoma, Pregnancy, Surgery, Estrogen receptor

## Abstract

**Background:**

Although spinal schwannomas generally grow very slowly, it has been reported that these clinical growths and their associated neurological symptoms accelerate during pregnancy. Because these cases are rare, surgical intervention for this tumor during pregnancy poses a significant challenge. The change of pregnancy-related hormones, such as estrogen and progesterone, is considered to have an effect on the clinical symptoms of spinal tumors. Expressions of the receptors for estrogen and progesterone in orbital and vestibular schwannomas have been reported; however, those expressions in spinal schwannomas have not been examined.

**Case presentation:**

A 36-year-old woman at 8 weeks' gestation suffered from developing neck pain and neurological symptoms in the right upper extremity. Magnetic resonance imaging (MRI) confirmed the presence of a cervical intradural extramedullary tumor. Under general anesthesia, using intraoperative neurophysiological monitoring of motor-evoked potentials (MEPs), spinal tumor resection following a hemi-laminoplasty was performed in a prone position at 12 weeks gestation. The pathological diagnosis following surgery was spinal schwannoma. Her neurological symptoms were significantly improved after surgery and she delivered a healthy baby in her 40th week of pregnancy. At a 12-month follow-up, no abnormalities were observed during medical examinations of both mother and child. An immunohistochemical study identified the expression of estrogen receptors, but not progesterone receptors, in the spinal schwannoma.

**Conclusions:**

A cervical spinal schwannoma was successfully removed under general anesthesia at 12 weeks gestation by coordination between orthopaedic, obstetric and anesthesia teams. For the first time, an immunohistochemical analysis showed that the expression of estrogen receptors was identified in spinal schwannoma cells, suggesting the possibility that these hormone receptors in spinal schwannoma might contribute to the worsening of neurological symptoms during pregnancy.

## Background

Spinal schwannomas are benign nerve sheath tumors within the spinal canal, typically arising from Schwann cells of dorsal nerve roots. They are the most common intradural extramedullary spinal tumor and represent approximately 55% of spinal tumors [1]. Spinal schwannomas during pregnancy are rare and surgical intervention of this tumor during this time poses a significant challenge. Although spinal schwannomas generally grow very slowly (with a growth rate of 5.3% per year [2]), it has been reported that its clinical growth and associated symptoms accelerate during pregnancy [3, 4]. Pregnancy-related hormones, such as estrogen and progesterone, are considered to be among the factors associated with this rapid growth [3, 5, 6]. Expressions of the receptors for estrogen and progesterone in orbital and vestibular schwannomas have been reported; however, those expressions in spinal schwannomas have not been examined.

The purpose of this study is (1) to report the case of a cervical schwannoma that was successfully resected in an early stage of pregnancy, and (2) to evaluate the immunohistochemical expression of receptors against pregnancy-related hormones in the spinal schwannoma.

## Case presentation

**Fig. 1:**
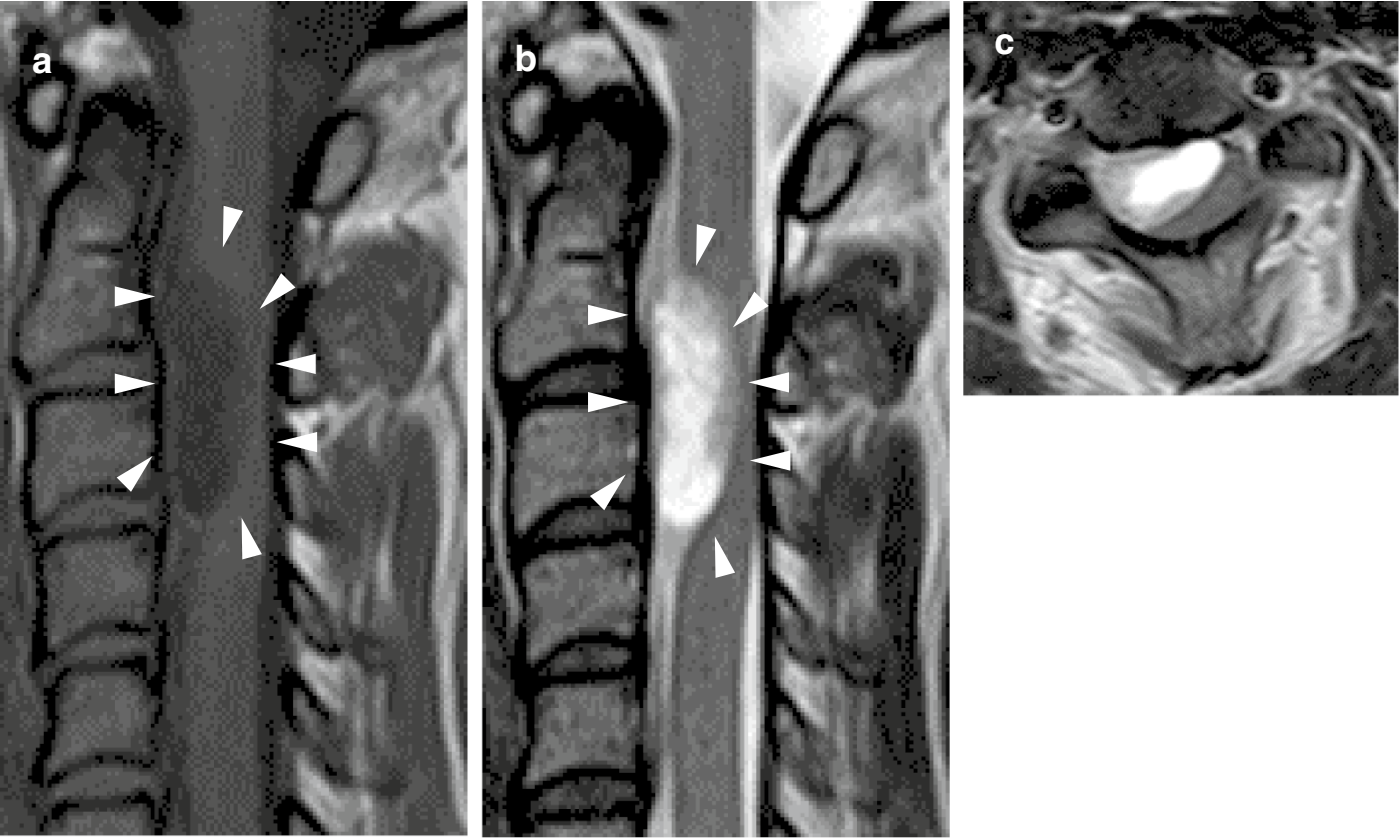
Preoperative magnetic resonance imaging (MRI) of the cervical spine. **a** mid-sagittal section of a T1-weighted image; **b** mid-sagittal section of a T2-weighted image; and **c** an axial T2-weighted image at the C3 vertebra. Arrowheads indicate the location of the spinal tumor

A 36-year-old woman at 9 weeks' gestation was transferred to our facility with severe posterior neck pain. She presented with a four month history of severe pain from the back of both ears to the ridge of the shoulder with symptoms gradually worsening at eight weeks of pregnancy. She also complained of numbness in fingers of both hands and difficulty in raising the right upper extremity. No significant muscle weakness in the lower extremities was identified. No unusual findings on cervical radiographs and computed tomography (CT) images were found. Magnetic resonance imaging (MRI) revealed an oval-shaped tumor (size: 30 × 15 mm) in the intradural space with a T1 – weighted image (WI) low and a T2 – WI high at the level from the C2 to C3 vertebrae. MRI showed that the spinal tumor severely compressed the spinal cord (Fig. 1). The initial diagnosis was an intradural extramedullary tumor.

**Fig. 2:**
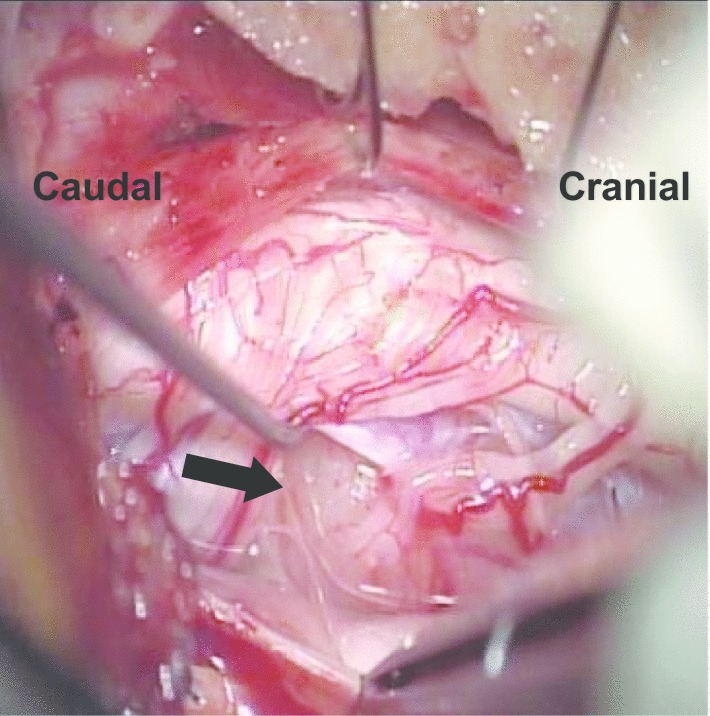
Intraoperative surgical findings under microscopy. An incision was made in the dura mater, and a tumor was confirmed on the anterolateral side of the spinal cord. The arrow indicates the spinal tumor

**Fig. 3:**
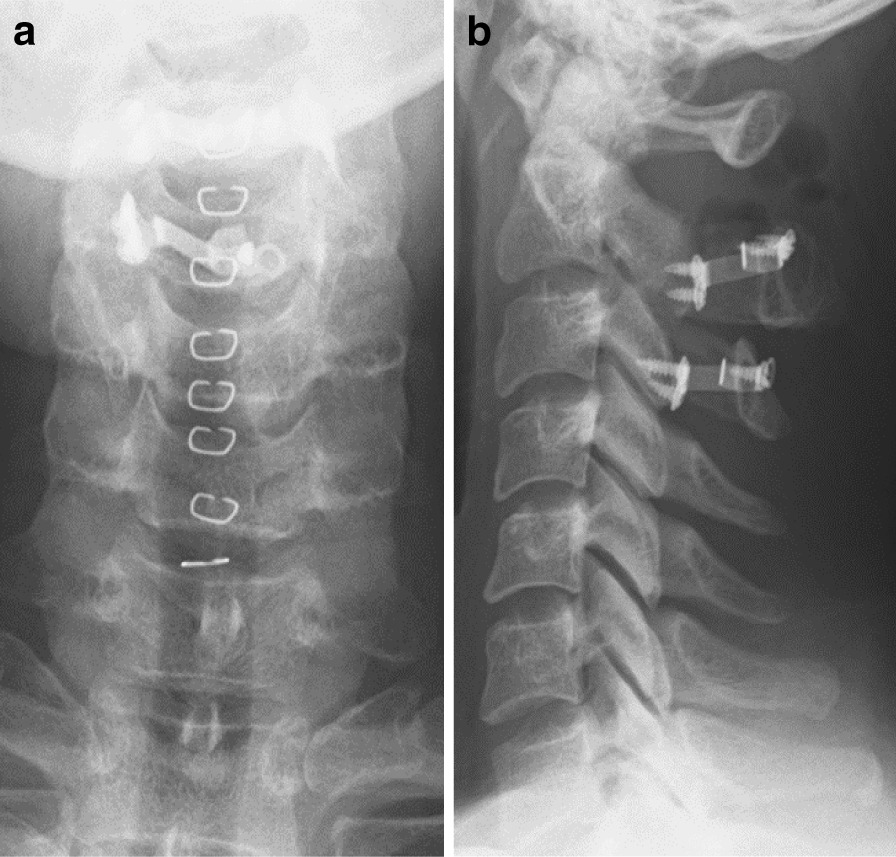
Postoperative cervical radiograph. **a** Anteroposterior view; **b** lateral view. A hemi-laminoplasty of C2 to C3 was performed, followed by placing an inter-lamina spacer (Centerpiece ®, Medtronics, USA)

Because of the patient's severe pain and progressing neurological symptoms, the patient felt that it was difficult to wait for the surgery until after delivery. The therapeutic strategy was determined by consulting with obstetric and anesthesia teams. Based on the results of consultations, the spinal cord tumor resection under general anesthesia at 12 weeks of gestation was planned. The patient and her family received sufficient explanation for the necessity of surgery, the method of general anesthesia, possible risks of the surgery, and complications. With an adequate understanding of the surgical treatment, the patient provided informed consent. Under general anesthesia with the use of intraoperative neurophysiological monitoring, a hemi-laminoplasty of C2 to C3 was performed in a prone position, and the spinal tumor was resected totally under microscopy (Fig. 2). Inter-lamina spacers (Centerpiece®, Medtronics, City, State, USA) were placed at the C2 and C3 lamina, followed by a dura suture (Fig. 3). Motor-evoked potentials (MEPs) were monitored continuously during surgery; no significant changes in MEP amplitude were observed.

**Fig. 4:**
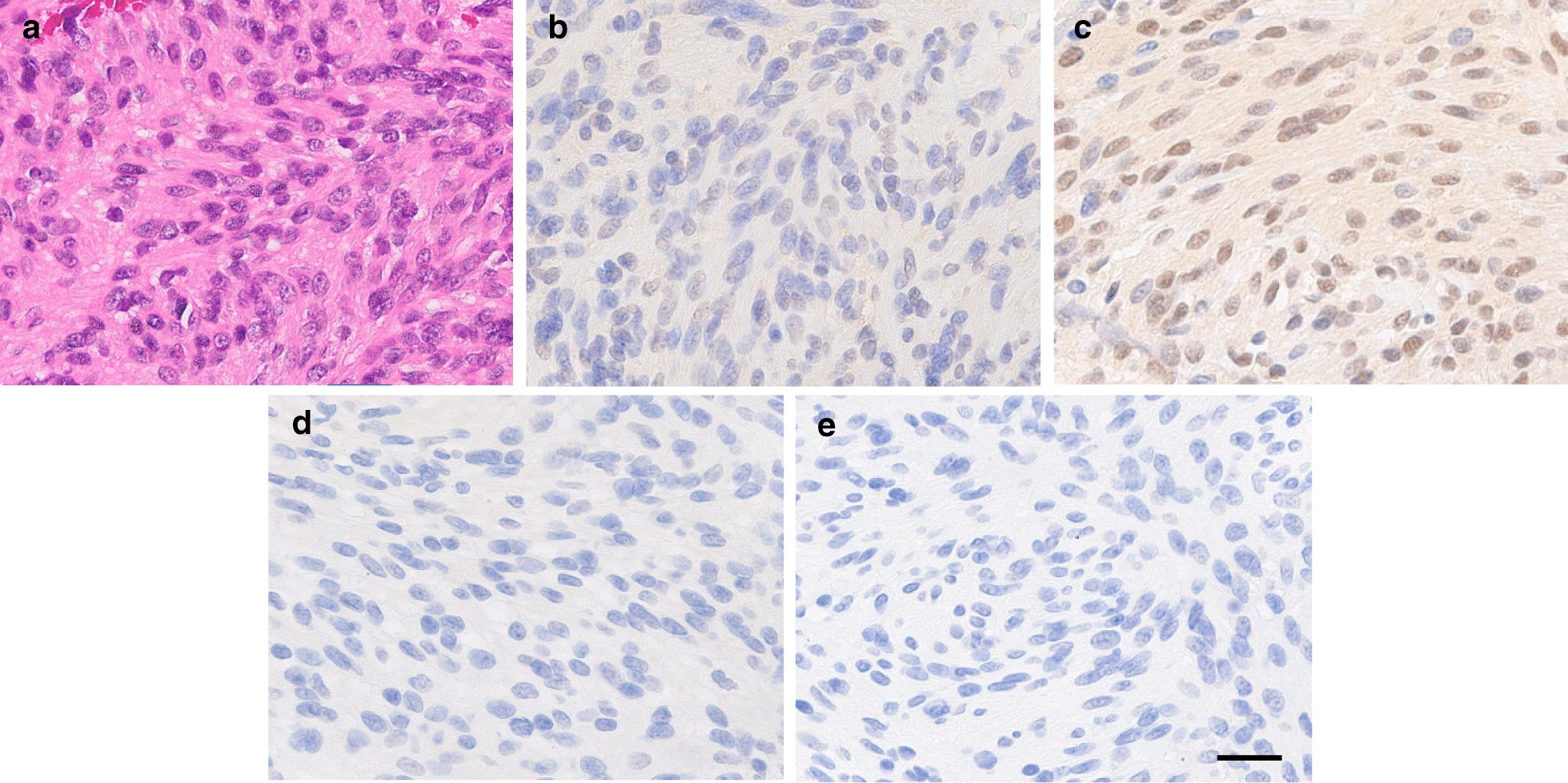
Immunohistochemical analysis. **a** Hematoxylin and eosin (H&E) staining of a cervical spinal schwannoma. Immunohistochemical staining for **b** estrogen α receptor; **c** estrogen β receptor; **d** progesterone receptor; and **e** isotype (negative) controls. Scale bar: 50 μm

**Fig. 5:**
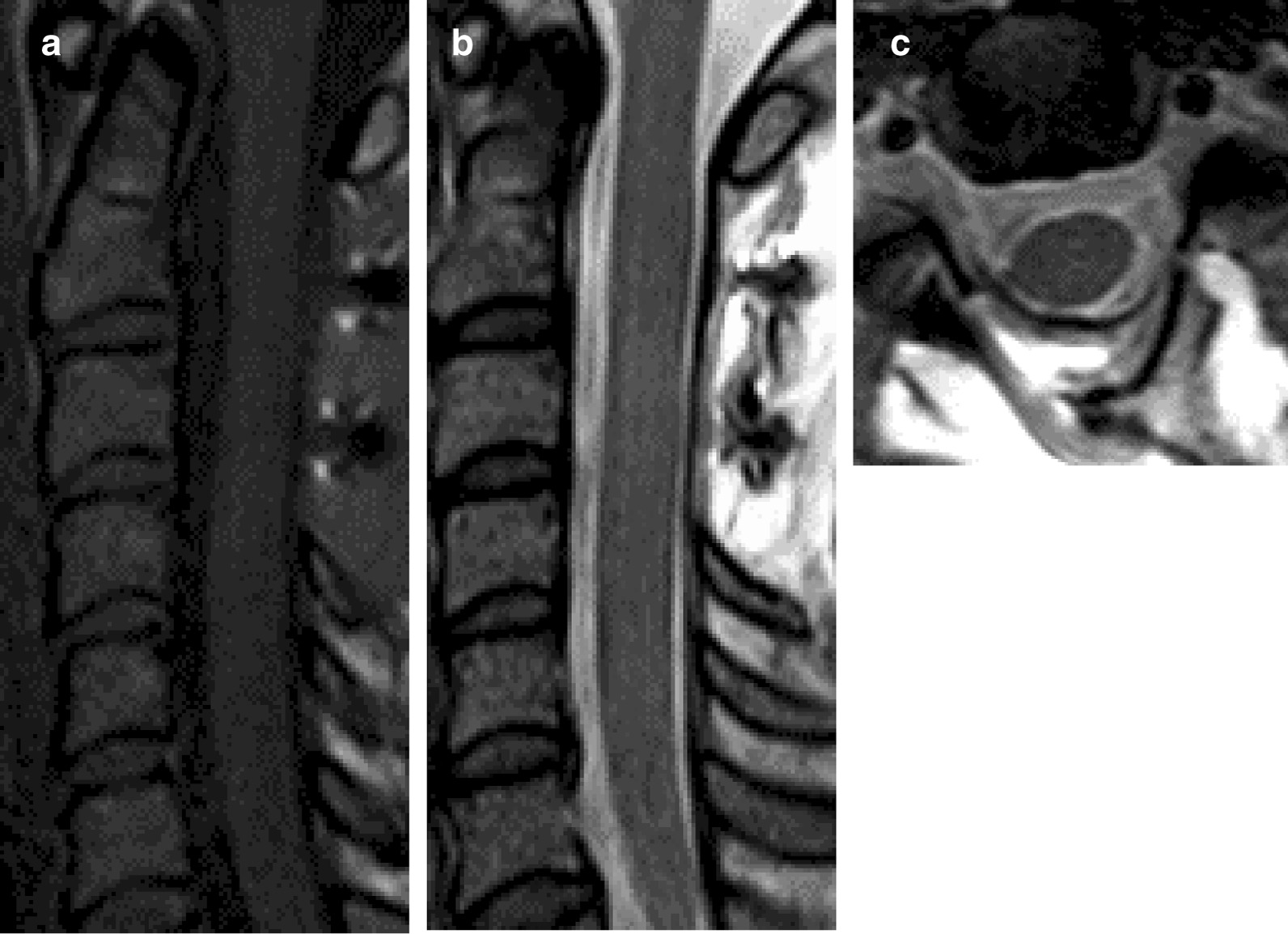
Postoperative magnetic resonance imaging (MRI) of the cervical spine. **a** Mid-sagittal T1-weighted image; **b** mid-sagittal T2-weighted image; and **c** axial T2-weighted image at the C3 vertebra

Histology (Hematoxylin & Eosin staining) of the removed tumor showed spindle-shaped cells in multiple directions in a myxoid collagenous background (Fig. 4a). The pathological diagnosis following surgery was schwannoma. The patient's neurological symptoms were significantly improved post-surgery. The post-operative MRI showed no residual tumor and the spinal cord was released (Fig. 5). The post-operative course was uneventful and the patient was discharged on post-operative day 14. Six months following the operation, she delivered a healthy baby in her 40th week of pregnancy. At a 12-month follow-up, the patient had no neck pain or neurological findings and no abnormalities were observed during medical examinations of both mother and child.

Immunohistochemistry was performed on formalin-fixed, paraffin-embedded tissues, as previously reported. In short, following endogenous peroxidase inactivation and heat-induced epitope retrieval, the sections were stained with estrogen receptor α antibody (M7047: Agilent, Santa Clara, CA, USA), estrogen receptor β (M7292: Agilent) and progesterone receptor (MA5-14505: Invitrogen, Carlsbad, California, USA). Mouse IgG (Agilent) was used as the isotype or negative control. The sections were visualized using the universal immuno-enzyme polymer method (Histofine Simple Stain MAX-PO; Nichirei Biosciences, Tokyo, Japan) and 3,3′-diaminobenzidine tetrahydrochloride (DAB; Dojindo, Tokyo, Japan), followed by counterstaining with Mayer's hematoxylin. Weak immunoreactivity against the estrogen α receptor was found on the nuclei and in the cytoplasm of schwannoma cells (Fig. 4b). On the other hand, intense immunoreactivity against the estrogen β receptor was clearly identified on the nuclei and in the cytoplasm of schwannoma cells (Fig. 4c). No significant immunoreactivity against the progesterone receptor was identified (Fig. 4d). No immunoreactivity was found in the isotype (negative) control (Fig. 4e).

## Discussion and conclusion

Spinal cord tumor resection was safely performed under general anesthesia for cervical spinal schwannoma at 12 weeks gestation in consultation with obstetrics and anesthesia teams. Immunohistochemical analysis revealed that immunoreactivity for the receptors of estrogen was identified in spinal schwannoma cells.

Because the coocurrence of spinal tumors and pregnancy is rare, the timing of surgery during pregnancy remains controversial [4]. Nossek et al. reported thirty-four cases of pregnant and early postpartum women who required a neurosurgical intervention [7]. Among these, sixteen patients underwent neurological intervention during pregnancy between 11 to 34 weeks of gestation. The authors concluded that intervention under general anesthesia is safe and should be considered early rather than late in most pregnant patients. Han et al. [8] retrospectively reviewed ten pregnant patients who underwent spinal surgery and reported that, in most cases, spinal surgery could be safely performed while maintaining pregnancy. They also suggested that the operation should be performed following induction of delivery for patients at 34 to 36 weeks gestation with deteriorating neurological symptoms. In our case, spinal surgery was successfully completed during the first trimester (at 12 weeks of pregnancy) because of progressive neurological symptoms.

Recently, to avoid spinal cord paralysis, the use of intraoperative neurophysiological monitoring has become essential in spinal cord tumor resection surgery. In this case, because the tumor strongly compressed the spinal cord, we needed to use motor-evoked potentials (MEPs) intraoperatively to prevent cervical cord paralysis during surgery. Thus far, three case reports on the use of MEPs for spine or brain surgery during pregnancy have been reported [9–11]. All three cases showed no deleterious intraoperative or postoperative complications in the mother and fetus with the use of MEP monitoring, suggesting the possibility that intraoperative spinal cord monitoring could be safely applied in pregnant women. Pastor et al. [11] reported a case report of a 34-year-old woman who was 26 weeks pregnant and required brain surgery. They utilized MEP and somatosensory-evoked potentials (SSEP) for intraoperative neurophysiological monitoring. Additionally, the uterine myometric tone of the mother and the fetal heart rate were also monitored. No remarkable changes related to electrical stimulation in either uterine muscle tone or fetal heart rate were observed, and no motor or new somatosensory deficits appeared. In our case, the fetal heart rate was not able to be monitored at 12 weeks of pregnancy because cardiotocography can only be used after 18 gestational weeks. The mother safely gave birth to the fetus in the 40th week of her pregnancy and no abnormalities were observed during medical examinations of both mother and child at a 12-month follow-up. Nevertheless, given that the safe use of MEPs during pregnancy has not been established, it would be necessary to carefully evaluate its use based on clinical symptoms, degree of spinal cord paralysis, and type of tumor.

In our case, the patient's neurological symptoms progressively worsened during the early stage of pregnancy. Therefore, the effect of pregnancy on spinal tumor growth was suspected. The mechanism of accelerated tumor growth during pregnancy remains unclear; however, two main mechanisms have been proposed [12]. First, increased blood volume due to pregnancy and the redistribution and increased blood flow volume through the vertebral venous plexus secondary to the gravid uterus compressing the vena cava contribute to tumor growth during pregnancy. Second, direct hormonal effects from progesterone receptors (PRs) and estrogen receptors (ERs) mediate tumor growth. Previous studies reported that pregnancy-related hormones could affect tumor growth and progression of neurological symptoms [5, 6, 13]. The results of previous immunohistochemical studies on the expression of PRs and ERs on orbital and vestibular schwannomas are summarized in Table 1. Immunoreactivity for PR was found in one report [6] and immunoreactivity for ER was also found in one report [14]. Patel and colleagues [15] reported the results of quantified mRNA expression of PR and ER in sporadic and neurofibromatosis 2 (NF2) vestibular schwannoma; they also showed differences in PR and ER mRNA expressions between the two types of tumors. These results suggest that the diversity in the pattern of PR and ER expression is dependent on the type of tumor.

**Table 1:** Immunohistochemical analysis of the expression of estrogen and progesterone receptors on orbital and vestibular schwannomas

Author	Type of tumor	Case	Staining	Antibody
Chang et al. [6]	Orbital schwannoma	1	PR: +	clone PGR 636 (Dako)
			ER: −	clone 1D5 (Dako)
Dalgorf et al. [16]	Vestibular schwannoma	1	PR: −	6F11 (Novocastra)
			ER: −	312 (Novocastra)
Hötte et al. [14]	Orbital schwannoma	2	PR: − (case 1 and 2)	clone SP107 (Ventana)
			ER: + (case 1), ER: − (case 2)	clone 1E2 (Ventana)

*PR* progesterone receptor, *ER* estrogen receptor

Because the expression of hormone receptors in spinal schwannoma had not yet been examined, we examined the immunohistochemical expression for ER and PR receptors. Our results showed that immunoreactivity to ER, but not PR, was identified in the spinal schwannoma in our case.

Furthermore, two distinct types of ERs (ERα and ERβ) have been shown to regulate cell growth distantly, proliferation, and differentiation in many cell types, including normal tissues (see review in [17]). ERα is well known to play an essential role in cell proliferation, especially in breast cancer cells in the presence of estrogen. On the other hand, EPRβ has been reported to inhibit ERα signaling in several types of cells [18, 19]. The results of our study showed that strong immunoreactivity to ERβ and weak immunoreactivity to ERα were identified in the spinal schwannoma cells. Future studies would be needed to examine the cellular functions of ER and PR in spinal schwannoma cells and their expressions during pregnancy.

## Conclusion

We have experienced the case of a cervical spinal schwannoma patient whose neurological symptoms progressively worsened during the early stage of pregnancy. In cooperation with obstetrics and anesthesia teams, the spinal tumor was safely removed under general anesthesia using intraoperative neurophysiological monitoring of MEPs at 12 weeks of gestation. Immunohistochemical analysis revealed the clear expression of estrogen receptor in the removed spinal schwannoma, which suggests that pregnancy-related hormones, such as estrogen, might contribute to the worsening of clinical symptoms in the case of our patient.

## Data Availability

This is a case report of a patient; to protect privacy and respect confidentiality, none of the raw data has been made available in any public repository. The original reports, laboratory data, images and clinic records are retained as per standard procedure within the medical records of our institution.

## Abbreviations

CT: Computed tomography
MRI: Magnetic resonance imaging
MEPs: Motor-evoked potentials
PR: Progesterone receptor
ER: Estrogen receptor
